# Characterization of Mucosal Dysbiosis of Early Colonic Neoplasia

**DOI:** 10.1038/s41698-019-0101-6

**Published:** 2019-11-14

**Authors:** Bo-young Hong, Takayasu Ideta, Bruno S. Lemos, Yuichi Igarashi, Yuliana Tan, Michael DiSiena, Allen Mo, John W. Birk, Faripour Forouhar, Thomas J. Devers, George M. Weinstock, Daniel W. Rosenberg

**Affiliations:** 1 The Jackson Laboratory for Genomic Medicine, Farmington, CT USA; 2 Department of Medicine, University of Connecticut Health, Farmington, CT USA; 3 Department of Gastroenterology and Hepatology, University of Connecticut Health, Farmington, CT USA; 4 Department of Pathology, University of Connecticut Health, Farmington, CT USA

**Keywords:** Predictive markers, Cancer prevention

## Abstract

Aberrant crypt foci (ACF) are the earliest morphologically identifiable lesions in the colon that can be detected by high-definition chromoendoscopy with contrast dye spray. Although frequently associated with synchronous adenomas, their role in colorectal tumor development, particularly in the proximal colon, is still not clear. The goal of this study was to evaluate the profile of colon-adherent bacteria associated with proximal ACF and to investigate their relationship to the presence and subtype of synchronous polyps present throughout the colon. Forty-five subjects undergoing a screening or surveillance colonoscopy were included in this retrospective study. Bacterial cells adherent to the epithelia of ACF and normal mucosal biopsies were visualized by in situ hybridization within confocal tissue sections. ACF showed significantly greater heterogeneity in their bacterial microbiome profiles compared with normal mucosa. One of the bacterial community structures we characterized was strongly correlated with the presence of synchronous polyps. Finally, using DNA mass spectrometry to evaluate a panel of colorectal cancer hotspot mutations present in the ACF, we found that three *APC* gene mutations were positively associated with the presence of *Instestinibacter sp*., whereas *KRAS* mutations were positively correlated with *Ruminococcus gnavus*. This result indicates a potential relationship between specific colon-associated bacterial species and somatically acquired CRC-related mutations. Overall, our findings suggest that perturbations to the normal adherent mucosal flora may constitute a risk factor for early neoplasia, demonstrating the potential impact of mucosal dysbiosis on the tissue microenvironment and behavior of ACF that may facilitate their progression towards more advanced forms of neoplasia.

## Introduction

Colorectal cancer (CRC) is the third leading cause of cancer-related deaths in the United States. Fortunately, the widespread application of screening colonoscopy, together with the identification and removal of precancerous polyps, has led to a significant overall reduction in CRC incidence.^1–3^ Despite the overall health benefits, endoscopic surveillance has failed to uniformly prevent the occurrence of CRC, particularly in the proximal colon.^4^ These limitations are underscored by patients who develop “interval” CRC between screening colonoscopies and subsequent surveillance. Most of these cases have been attributed to non-detected or incompletely resected proximal colon lesions that were initially present during the index colonoscopy.^5–8^ Thus, there is a need to develop more robust strategies that enable the accurate identification and removal of proximal colon lesions, and, importantly, to enable the identification of those individuals at increased risk for recurrent neoplasms at the time of index colonoscopy.^9^

Aberrant crypt foci (ACF) are the earliest morphologically detectable lesions that are frequently present in the colon.^9^ However, ACF are not routinely detected during conventional colonoscopy due to their diminutive size (<5 mm in diameter).^10–14^ Despite evidence that ACF are often associated with the presence of adenomas, their role in tumor development, particularly in the proximal colon, is still actively debated.^15–18^ Our laboratory has recently demonstrated the power of high-definition chromoendoscopy to identify colonic ACF within the proximal colon.^19^ We further validated an ultra-sensitive DNA mass spectrometry platform that, combined with laser-capture microdissection, enables the detection of somatic mutations across a wide panel of CRC-related oncogenes and tumor suppressor genes.^9,19^ We believe these recent studies, including our genome-wide methylation analysis of ACF,^20^ firmly establish the premalignant potential of ACF and their potential application to risk prediction. In particular, we have focused on proximal colon ACF, which we believe may harbor greater premalignant potential.^9,19^

During the past two decades, there has been a growing appreciation for the role of the gut microbiome in CRC pathogenesis.^21^ *Fusobacterium nucleatum* was identified as an abundant taxon from colorectal tumor tissues, whereas other species were also detected.^22,23^ *Escherichia coli* has also been associated with CRC. Colonic biopsies of adenomas and CRCs showed the increased presence of intracellular *E. coli*,^24^ whereas higher abundance of other bacterial species of the phylum Proteobacteria were found in rectal adenomas.^25^ Enterohemorrhagic *E. coli* and enteropathogenic *E. coli* are also known to be risk factors for CRC, most likely by generating toxins that affect colonic tissue.^26,27^ Toxin-producing *Bacteroides fragilis* can impact colorectal carcinogenesis by its production of *B. fragilis* toxin, resulting in the disruption of E-cadherin junctions, β-catenin signaling, and interleukin-8 expression.^28,29^

Our understanding of the characteristics of the colon-associated microbiome and its potential role in the development of early colonic neoplasia remain incomplete. The objective of this study is to characterize the microbiota directly associated with proximal colon ACF. Our long-term goal is to determine whether the microbiome can influence the microenvironment and behavior of early colonic neoplasia, either facilitating (or impeding) the progression of small lesions to more advanced forms of neoplasia.

## Results

### Description of Clinical and Demographic Information

For this study, we retrospectively selected a total of 45 patients who had undergone a routine screening colonoscopy at the John Dampsey Hospital (JDH). We assigned the patient samples to the following three experimental groups: Group I (*n* = 16 patients) had no identifiable lesions (ACF or polyps) present in the proximal colon; Group II (*n* = 14) had at least one proximal ACF detected at colonoscopy, but no synchronous polyps; Group III (*n* = 15) had at least one or more proximal ACF and synchronous polyp(s) detected at the time of colonoscopy (Supplementary Table 1). The characteristics of the study population are shown in Table 1.

**Table 1:** Clinical and demographic characteristic of subjects included in the study

Demographics	Normal		ACF only		ACF + Polyp	
Sample size	16	(35.5)	14	(31.1)	15	(33.3)
Age	55.9 ± 9.9		55.9 ± 7.5		57.5 ± 7.7	
Male	8	(50.0)	3	(21.4)	8	(53.3)
BMI (kg/m^2^)	30.0 ± 7.0		29.5 ± 6.7		30.9 ± 6.8	
Caucasian	9	(56.3)	10	(71.4)	14	(93.3)
African American	7	(43.8)	4	(28.6)	1	(6.7)
Smoking history	9	(56.3)	6	(42.9)	9	(60.0)
Current smoker	3	(18.8)	2	(14.3)	3	(20.0)
Family history of colon cancer	5	(31.3)	6	(42.9)	3	(20.0)
Diabetes	4	(25.0)	3	(21.4)	2	(13.3)
Daily aspirin (325 mg) use	5	(31.3)	3	(21.4)	5	(33.3)
Daily baby Aaspirin (81 mg) use	6	(37.5)	4	(28.6)	6	(40.0)
Regular other NSAIDs use	5	(31.3)	8	(57.1)	7	(46.7)
Regular multivitamin use	9	(56.3)	9	(64.3)	4	(26.7)
Regular folic acid use	3	(18.8)	1	(7.1)	1	(6.7)
Regular calcium use	3	(18.8)	6	(42.9)	3	(20.0)
Regular vitamin D use	3	(18.8)	1	(42.9)	7	(46.7)
Antibiotics use (within 1 month)	1	(6.3)	1	(7.1)	0	(0.0)

Values are means ± SD or *n* (%)

### Direct visualization of colon-associated bacteria in the colonic mucosa

**Fig. 1:**
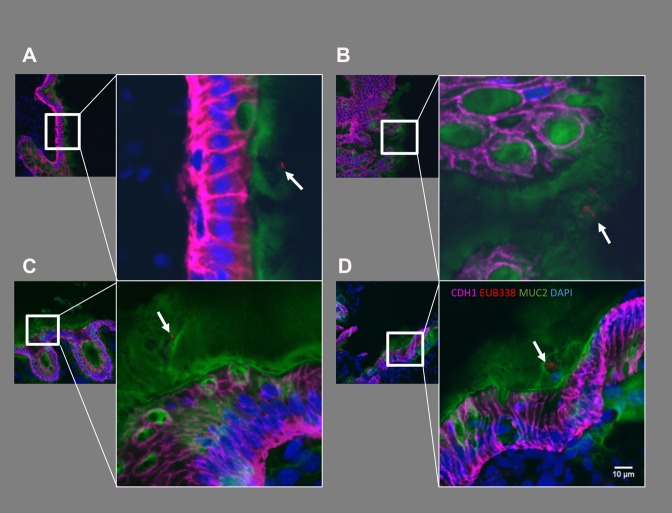
Direct visualization of tightly colon-associated bacteria within the colonic mucosa. Proximal ACF and adjacent normal mucosa were directly examined for the presence of colon-associated bacteria using 16S universal FISH probes. Clusters of bacteria were observed within the mucous layer on epithelium. Red: bacteria (EUB338-cy3 probe), Magenta: E-cadherin, Green; Mucin-2, Blue: DAPI. Bacteria are marked with white arrows. Bacteria were observed within the mucous layer associated with the epithelium

ACF, adjacent normal mucosa, and synchronous polyps (tubular adenomas, hyperplastic polyps (HPs), and sessile serrated adenoma polyps (SSA/P)) in Group III were directly examined for the presence of adherent bacteria using 16S universal fluorescence in situ hybridization (FISH) probes. Representative lesions are shown in Fig. 1 (ACF and adjacent normal) and also in Supplementary Fig. 1 (synchronous polyps identified in Group III). Bacteria were observed within the mucous layer (green; mucin-2 positive) of the colonic epithelial lining in normal, ACF, and polyp tissues. However, in no cases did we observe bacterial infiltration beyond the mucosal layer and into the colonic epithelial lining, nor within the stroma (Fig. 1 and Supplementary Fig. 1). In addition, colon-associated bacteria showed no detectable differences morphologically.

### Analysis of Proximal ACF and Colon-Associated Microbiome

Illumina sequencing of the V4 hypervariable region of 16S rRNA amplicons from all individual samples yielded 7,469,049 raw reads and 4,414,345 reads after pre-processing of the data set. The sequence counts per sample ranged from ~5627 to 319,577 reads. Libraries were normalized by random sub-sampling for comparisons across the samples. In total, 815 operational taxonomic units (OTUs) were found at a 97% identity cutoff from 74 samples. In order to determine α- and β-diversity, we compared the adherent bacteria of ACF with adjacent normal colonic mucosa from patients with or without polyps, separately. We also compared adherent bacteria of ACF with the normal colonic mucosa of 16 control patients who had no detectable proximal ACF.

First, α-diversity analysis (Supplementary Fig. S2A) showed a mean richness of 106, 111.9, 105, 131.6, and 120.2 in the normal mucosa from Group I, normal mucosa from Group II, a proximal ACF from Group II, normal mucosa from Group III, and a proximal ACF from Group III, respectively. Proximal ACF lesions and adjacent normal mucosa from patients with synchronous polyp(s) showed median richness of 103.3 and 99.6 distinct bacterial taxa, respectively. These results were slightly lower than median richness of 110 bacterial taxa found in normal mucosa from the lesion-free patients. However, there was no statistically significant difference in bacterial richness between these groups. In addition, diversity and evenness of the bacterial community within each sample showed no statistically significant differences (Supplementary Fig. S2B, C). Nonetheless, subject variability of the bacterial microbiome in lesions from patients with a polyp was consistently greater than lesion-free patients for richness, diversity, and evenness.

**Fig. 2:**
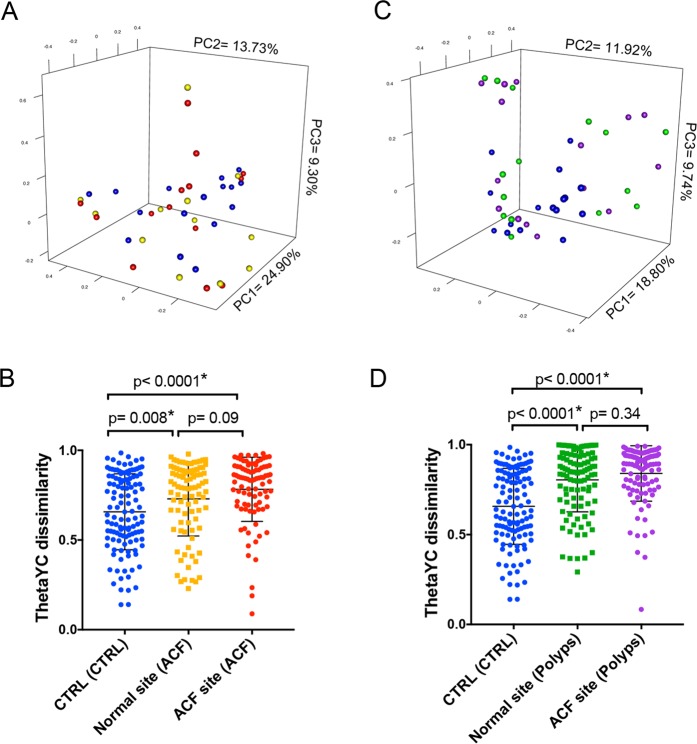
β-Diversity comparison of the microbiome between normal mucosa and ACF based on thetaYC distance. In polyp-free subjects, principle coordinate analysis (PCoA) plots showed no significant spatial separation as determined by AMOVA between proximal ACF lesions and control mucosa taken from both ACF-free control and paired normal samples from the same ACF subject **a**. The distance measured pairwise between each sample, however, showed significant differences in microbiome community structure **b**. In polyp subjects, PCoA plots showed no significant spatial separation tested using AMOVA between proximal ACF lesions and control mucosa from both ACF-free controls and paired normal samples taken from the same ACF subject **c**. However, ACF samples from patients with polyps were better rsolved from control samples (blue) compared with that in **a**. In addition, the distance measured pairwise between each sample showed significant differences in microbiome community structure **d**, with more significant *p*-values from **b** when comparing the ACF site from polyp subjects (purple) to control group (blue) using Mann–Whitney test

Next, we characterized the bacterial community structure of ACF biopsies taken from the proximal colon with or without synchronous polyp(s) (Fig. 2). Pairwise comparison of bacterial community structure based on the thetaYC index, which takes into account membership of taxa and their relative abundance, was measured between samples. Bacterial community structure visualized in principal coordinates analysis (PCoA) plots showed no distinct clustering of proximal ACF compared with their normal adjacent mucosa or normal mucosa from ACF lesion-free patients (*p* = 0.139) (Fig. 2a). However, there was a trend observed in which control mucosal samples (blue) clustered more tightly than ACF samples (red) or their adjacent normal mucosa (yellow), suggesting that normal mucosa is somewhat more homogeneous in community structure between samples than the ACF lesions in terms of bacterial diversity (Fig. 2a). Furthermore, we confirmed that the distance measured from pairwise thetaYC dissimilarity comparisons between every sample in proximal ACF (purple) and their adjacent normal mucosa (green) were both significantly different from that of control normal mucosa (blue) (*p* < 0.0001, *p* = 0.008) (Fig. 2b), indicating that ACF patients harbor a significantly more heterogeneous bacterial structure than control subjects with no detectable lesions. These results indicate that bacterial community structure has been significantly altered in proximal ACF of Groups II and III.

Next, to determine whether microbiome changes are associated with an at-risk colon, we analyzed a subset of proximal ACF and adjacent normal mucosa from patients with synchronous polyp(s). Interestingly, the PCoA plot showed a clearer tight clustering of control mucosal samples (blue) from ACF samples (purple) or their adjacent normal mucosa (green) (*p* = 0.004) (Fig. 2c). Microbiome alterations that were observed in proximal ACF samples with synchronous polyp(s) were greater than those changes found in ACF samples without synchronous polyps. The thetaYC dissimilarity pairwise comparison between samples confirmed a significant difference between normal mucosa and proximal ACF with synchronous polyp(s) (*p* < 0.0001) (Fig. 2d), implicating mucosal dysbiosis in the at-risk colon.

### Proximal ACF-Adherent Bacterial Taxa are Stratified by Synchronous Polyps and Somatic Mutations

We further identified differentially abundant specific bacterial taxa present within ACF lesions from patients with and without synchronous polyp(s) (Supplementary Fig. S3). The top 15 most differentially abundant taxa in ACF lesions taken from patients without synchronous polyps included a significant increase of *Lactonifactor sp*. (OTU036) and *Pantoea sp*. (OTU038), as well as a significant decrease of *Clostridium XIVa sp*. (OTU103); however, each of the 15 taxa yielded a relative abundance of <1%. On the other hand, the 15 most differentially abundant taxa present in ACF lesions taken from patients with synchronous proximal polyps included a significant increase of *Eubacterium sp*. (OTU060) and *Clostiridium sensu stricto sp*. (OTU087). Notably, the levels of *Faecalibacterium sp*. (OTU001) were significantly lower in mucosal samples taken from patients without synchronous polyps compared with normal mucosa from control subjects.

**Fig. 3:**
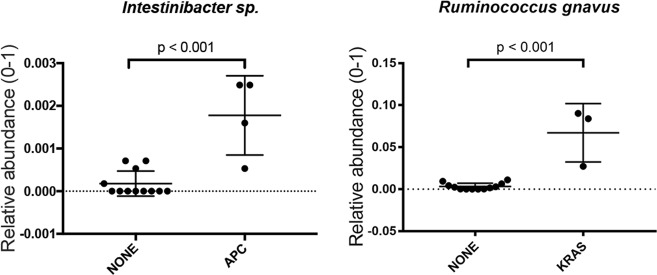
Significantly abundant bacterial taxa by DNA mutation types. *Intestinibacter sp*. was significantly increased in ACF samples with an *APC* mutation, whereas *R. gnavus* was significantly increased in ACF samples with a *KRAS* mutation compared with samples with no mutation detected

In the following analysis, we determined whether the presence of a somatic mutation in proximal ACF may influence the association and composition of colon-associated bacterial species. To test this possibility, host DNA was examined by DNA mass spectrometry analysis using our recently reported customized CRC mutation panel.^9^ Patients harboring a somatic mutation to the tumor suppressor gene, *APC* (R1450*, R876*, S1465fs*3), showed a significant correlation with *Intestinibacter sp*. (*p* < 0.001). ACF with a mutation in the proto-oncogene, *KRAS* (G12V, G12D*)*, showed a significant correlation with *Ruminococcus gnavus* (*p* < 0.001) (Fig. 3). Although this analysis is limited in sample size, these results provide the specific indication of a potential relationship between specific adherent bacterial species and a particular CRC-related somatic mutation.

### Correlation of Proximal ACF Bacterial Communities and Clinical Parameters

**Fig. 4:**
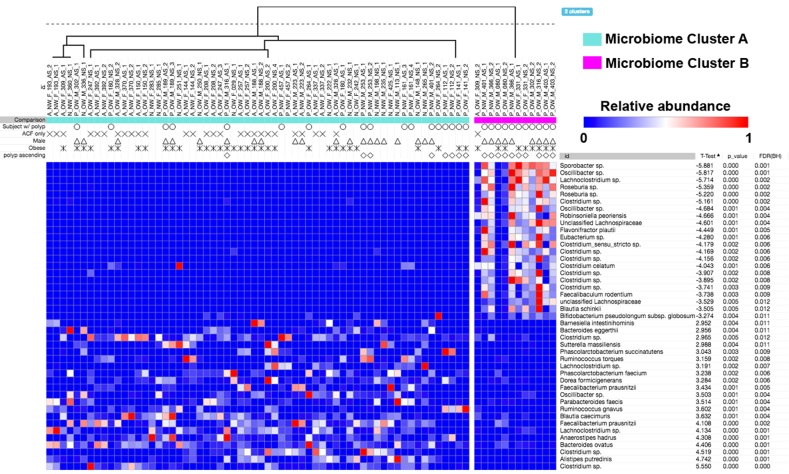
Microbiome profiles of the entire sample set. Heat maps were generated using Morpheus based on the top 100 OTUs found in the biopsy specimens. Samples are shown in columns, while each OTU is depicted in the rows. Only significantly different taxa in each cluster are shown. The color scale appears on the top right side of the figure. Unsupervised hierarchical clustering (complete linkage) shows two clusters, Microbiome Cluster A (cyan) and Microbiome Cluster B (magenta). Annotation for the presence of a polyp(s) in the subject, gender, and obesity, as well as the presence of a polyp(s) in the ascending colon are indicated by unique shapes when positive. Significance was determined after a Benjamini–Hochberg multiple comparison adjustment

We further investigated the extent to which changes in the microbiome community signature of proximal ACF may be associated with polyp incidence SSA/P, HPs or conventional adenomas. Figure 4 shows ACF bacterial profiles depicted as a heat map, with gender and body mass index (BMI) of the patients denoted. Two distinct microbiome clusters segregated by bacterial community profile were identified in proximal ACF lesions; 62 samples were present in Microbiome Cluster A and 12 samples were present in Microbiome Cluster B. The presence of a proximal ACF lesion alone did not distinguish Microbiome Clusters A and B (*p* = 0.4089). However, a proximal ACF lesion with or without the presence of a synchronous polyp clearly distinguished Microbiome Clusters A and B (*p* < 0.0001), indicating that a distinct bacterial community composition may be associated with an at-risk colon. Importantly, >50% of proximal ACF samples within Microbiome Cluster A occurred in the absence of synchronous polyps, whereas >90% of proximal ACF samples within Microbiome Cluster B had at least one synchronous polyp present in the same subject. The top five differentially abundant OTUs were taxonomically classified as *Clostridium sp*., *Alistipes putredinis*, *Clostridium sp*., *Bacteroides ovatus*, and *Anaerostipes hadrus* (Microbiome Cluster A), whereas *Sporobacter sp*., *Oscillibacter sp*., *Lachnoclostridium sp*., *Roseburia sp.*, and *Roseburia sp*. are Microbiome Cluster B.

**Fig. 5:**
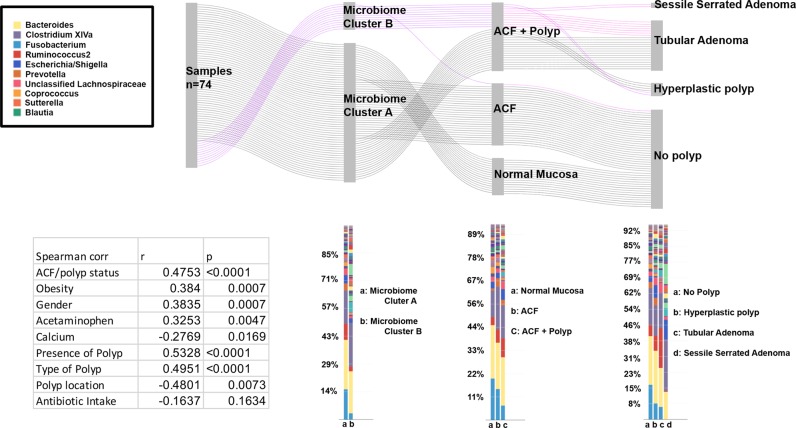
Microbiome signature shapes the development of polyps. Two distinct microbiome clusters (Microbiome Clusters A and B) defined by bacterial community signature were depicted in the Sankey diagram, demonstrating the “flow” of each biopsy sample. Microbiome Cluster B is associated with precancerous lesions. Over 90% of samples with Microbiome Cluster B yielded malignant polyps in the same subject. Less than 50% of samples with Microbiome Cluster A were associated with the presence of any polyp in the same subject (Top). Bacterial relative abundances in each category were depicted in the bar graph comparing groups from the Sankey diagram above. There were bacterial community differences by Microbiome Cluster type, ACF/polyp status (no lesion, ACF, and ACF with polyp), and the specific type of polyp present in the subjects. Microbiome Clusters A and B were significantly correlated with demographic or clinical characteristics, such as obesity status, gender, acetaminophen, and calcium intake. Antibiotic intake was not significantly correlated with Microbiome Clusters A and B (Table 1)

Based on the strong association for the presence of a synchronous polyp(s) with Microbiome Cluster B, we further investigated the location and subtype of polyps (Supplementary Table 2) associated with this microbiome cluster (Fig. 5). Out of the 15 subjects represented within this group (Group III), 1 subject had a proximal SSA/P, 10 subjects had tubular adenoma(s), and 4 subjects had HPs, whereas 1 sample (Group II) from Microbiome Cluster B had no evidence of a synchronous polyp. Overall, Microbiome Cluster B was significantly correlated with the presence of a colon polyp (*p* = 0.0073). Furthermore, all normal mucosal samples were found within Microbiome Cluster A. Although our sample sizes are somewhat limited, we believe these results suggest that the Microbiome Cluster B profile may be associated with the presence of mucosal dysbiosis.

As further shown in Fig. 5, known CRC risk factors, including gender, obesity, and polyp location were significantly correlated with Microbiome Cluster B (*p* = 0.0007, *p* = 0.0007, *p* = 0.0073 for these three risk factors, respectively). These results suggest that the distinct microbiome clusters observed in this study are in concordance with a combination of CRC risk factors. Interestingly, acetaminophen intake was positively correlated with Microbiome Cluster B (*p* = 0.0047), whereas calcium consumption was negatively correlated with Microbiome Cluster B (*p* = 0.0169). It is noteworthy that antibiotic intake did not significantly correlate with either Microbiome Cluster A or B (*p* = 0.163).

### Predictive Metabolic Function

**Fig. 6:**
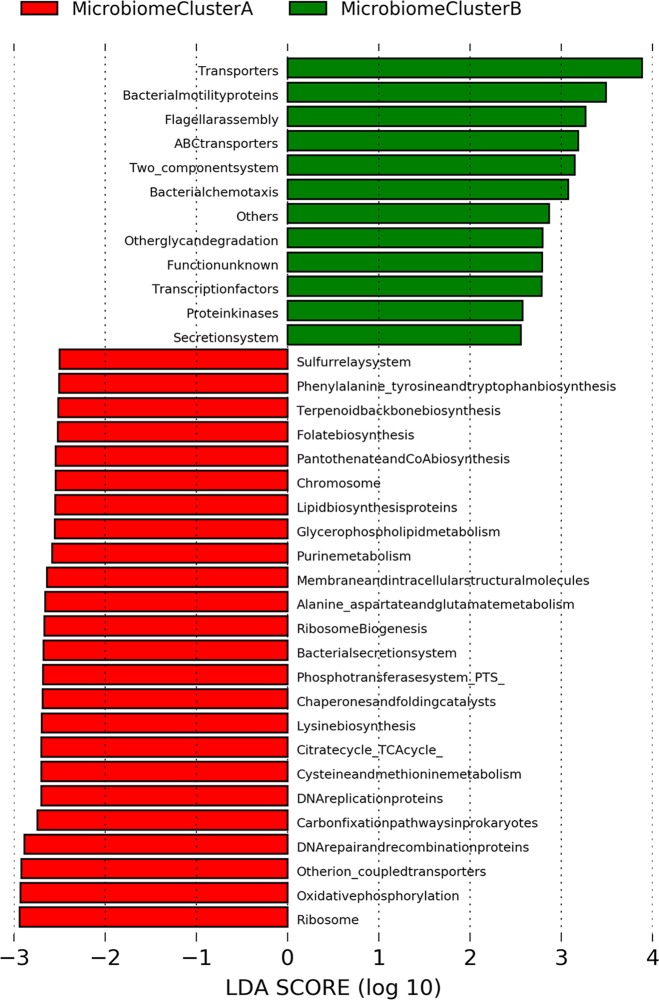
Predictive functional profiling annotation between Microbiome Clusters A and B. 16S rRNA gene sequencing data were clustered as OTUs with 97% identity and predictive functional profiling was performed via PICRUSt using KEGG KO as a reference. Several virulence factors were predicted as functional pathways associated with Microbiome Cluster B (green)

Several functional Kyoto Encyclopedia of Gene and Genomes (KEGG) pathways of potential clinical significance were predicted within bacterial communities present between Microbiome Clusters A or B (Fig. 6). Microbiome Cluster A showed functional changes associated with lipid metabolism, amino acid metabolism, nucleic acid metabolism, carbohydrate metabolism, and metabolism of co-factors and vitamins, as well as DNA replication, repair, folding, sorting and degradation, and translation. On the other hand, Microbiome Cluster B was associated with the following predicted pathways: cell motility, membrane transport, and signal transduction (Fig. 6).

## Discussion

In this study, we characterized the microbiome associated with early colonic neoplasia. We determined the composition of the colon-associated microbiome in proximal ACF and its relationship to the presence of synchronous polyp(s) present within colons of normal colonoscopy screening subjects. Our long-term goal is to uncover specific microbiota–epithelial interactions and to further understand how microbial dysbiosis may impact the microenvironment of the colon. It is likely that specific microbiota–epithelial interactions may directly influence the growth potential of proximal colonic ACF. The present study begins to develop a comprehensive microbiome data set of precancerous lesions present in the proximal colon, including their histological features and associated somatic mutations that can be combined with detailed information on microbial community structure. We believe this combinatorial approach will enable a sensitive and comprehensive prediction of early cancer risk and may ultimately provide new avenues for cancer prevention strategies based upon the targeted manipulation of the gut microbiota.

*F. nucleatum* has been identified in a number of studies as one of the most abundant bacterial species associated with CRC tumors.^22,30–34^ Our data indicate that precancerous proximal ACF also display evidence of dysbiosis of colon-associated mucosal bacteria. However, the specific taxa shown to be associated with advanced neoplasia, such as the abovementioned *F. nucleatum*, was neither prevalent nor abundant in our sample cohort. We believe our findings are consistent with those of a study^35^ showing that *F. nucleatum* stimulates the growth of colorectal cancer cells via interactions with the FadA receptor without directly affecting adenomatous tissue, leading the authors to speculate that *F. nucleatum* may provide a “second hit” to the initiated colonic epithelium. Regardless, our results indicate significant dysbiosis of mucosal adherent microbiota, with an increase in presumed pathogens and a decrease in a number of taxa that may have beneficial properties. This latter group of microbiota may also include a significant decrease of *Faecalibacterium prausnitzii*, one of the more abundant taxa shown to have anti-inflammatory activity in the human intestine.^36,37^ This is also consistent with a recent meta-analysis of mucosal microbiota, in which *Faecalibacterium* was reported to be significantly decreased in polyp tissues and CRC.^38^ Overall, our results suggest that dysbiosis of the colonic mucosal microbiome is associated with the more advanced forms of colonic neoplasia.

Our results further demonstrate that it is possible to provide a detailed view of tightly associated microbial composition obtained from even the smallest mucosal biopsy specimens (~2 mm^3^), even after a thorough pre-colonoscopy purging protocol. Although it is likely to be that luminal bacteria have a more complex community structure, in this study we have focused exclusively on tightly adherent mucosal microbiota. Mucosal adherent bacteria are likely to play a more direct role in the pathogenesis of CRC than luminal bacteria.^39^ Thus, we believe that this bacterial pool may provide greater insight into mucosal-specific colonic neoplasia due to their close interactions with epithelial and immune cells despite their lower overall bacterial load and complexity. Our sample set has readily provided several distinct microbial clusters, referred to as Microbiome Clusters A and B, showing a clear distinction among the three groups. Microbiome Cluster B has a particularly strong association with the presence of synchronous polyps in the colon, including SSAs, TAs, and HPs. We further identified a group of taxa strongly associated with Microbiome Cluster A.

Based upon our in silico analyses, we are aware of the relatively low taxonomic resolution that has resulted from the V4 hypervariable region of the 16S rRNA gene (data not shown). In fact, many of the OTUs identified by our analysis were classified at the genus level without inclusion of species names. However, the present study provides the specific OTU-level bacterial community analysis associated with early neoplastic changes occurring within the human colonic mucosa.

Predictive metabolic function analysis demonstrated that the functional categories associated with Microbiome Cluster B comprised a group of virulence factors; these include bacterial motility proteins and additional proteins associated with the two-component system, secretion system, flagella assembly, and chemotaxis. These results are consistent with a previous study that demonstrated many similar predicted metabolic pathways that were reportedly enriched in colorectal tumors.^40^ The metabolic functions represented in Microbiome Cluster B may implicate bacterial community composition and their biological markers, and this may further explain the extent of mucosal dysbiosis uniquely observed in Microbiome Cluster B, even in the earliest precancerous ACF lesions.

To gain further insight into how these microbial–epithelial interactions may impact early colonic neoplasia, we performed an analysis of CRC-related somatic mutations on a subset of laser-captured ACF samples using our customized CRC somatic mutation panel and DNA mass spectrometry.^9^ We found that *Intestinibacter sp*. and *R. gnavus* are strongly associated with the presence of either an *APC* or *KRAS* mutation, respectively (Fig. 3). These results provide what we believe is the direct evidence that cancer-related somatic mutations present at the earliest stages of colonic neoplasia may be directly associated with the presence of specific microbial organisms. Although these findings represent only a limited sample size, they do raise the possibility of a direct host–microbial interaction within the colonic mucosa that may contribute to neoplastic progression, findings we believe warrant further investigation.

In summary, our study suggests the comprehensive characterization of a distinct microbiome profile directly associated with proximal ACF, the earliest morphologically identifiable lesion in the colon that likely precedes the formation of more advanced forms of colonic neoplasia. We believe that our emphasis on the characteristics of the microbiome during early neoplasia will have a significant impact in terms of clinical application. Capturing integrated and combined characteristics of precancerous lesions, ranging from somatic mutations, histology, and microbiome composition, will eventually lead to a more sensitive and comprehensive detection of cancer risk, and contribute to efforts in cancer prevention and advanced colonoscopy screening based on the gut microbiome.

## Methods

### Study Population and Colonoscopy Procedure

Eligible healthy adults (50–65 years of age) that were referred to the Division of Gastroenterology at the John Dempsey Hospital/University of Connecticut Health (Farmington, CT) for screening or surveillance colonoscopy were recruited to the ACF study by participating physicians during their initial office consultation. Patients were screened for a standard-of-care colonoscopy procedure. Mucosal biopsy samples were procured under strict guidelines approved by the Institutional Review Board from the University of Connecticut Health Committee (#IE-10-068SJ-3.2) between 2010 and 2017. All participants provided written informed consent prior to inclusion in the study. Patients who met the Amsterdam criteria for familial adenomatous polyposis or hereditary non-polyposis CRC were excluded. Patients with ulcerative colitis, active infectious gastroenteritis, proctitis, diseases of malabsorption, or a history of CRC were also excluded. To limit the confounders of age and smoking, all subjects selected were non-smokers. Prior to colonoscopy, all participants completed a study questionnaire, including information on smoking, current medications and supplement use, previous history of endoscopy, and family history of cancer. BMI (kg/m^2^) was calculated from weight (kg) and height (m) measurements obtained during the initial office consultation.

High-definition chromoendoscopy was performed in the distal 20 cm of the colorectum and throughout the entire proximal colon with a freshly prepared solution of 0.1% indigo carmine dye spray, using a spray catheter for contrast enhancement. The identification and histologic evaluation of ACF has been described in our previous publications.^9,41^ Proximal ACF were isolated from grossly normal-appearing colonic mucosa by biopsy in situ and were removed using biopsy forceps. In addition, each subject had a histologically confirmed corresponding normal biopsy specimen taken from the same (proximal) region of the colon, generally within 2 cm of the ACF biopsy sample. Proximal ACF were identified if two or more crypts had an increased luminal diameter of 1.5 to 2 times the luminal size of surrounding crypts. Proximal ACF were visualized, videotaped, and photographed using an Olympus high-definition colonoscope. Biopsies of individual proximal ACF and/or normal colonic mucosa were embedded immediately in optimal cutting temperature (OCT) media and stored at −80 °C until further analysis. Samples were stained with hematoxylin and eosin, and ACF and polyps were histologically confirmed by a board-certified gastrointestinal (GI) pathologist (F.F.).

### Confocal Microscopy

Human tissues were stained by fluorescence in situ hybridization using a 16S rRNA probe as previously described.^42^ Briefly, OCT-embedded tissue was cryo-sectioned at a 5 µm thickness and then fixed in 4% paraformaldehyde for 24 h at 4 °C. Formalin-fixed paraffin-embedded human polyp tissues were de-paraffinized with sequential washes in xylene and ethanol. Samples were then hybridized using the EUB338-cy3 probe (Eurofins Genomics) at 55 °C overnight. Immunofluorescence was performed with 5% BSA blocking, followed by staining with anti-Mucin-2 (Santa Cruz Biotechnology) and anti-E-cadherin (Cell Signaling Technology) at 4 °C overnight. Alexa Fluor 488 (Thermo Fisher) and Alexa Fluor 647 (Thermo Fisher) were used to detect Mucin-2 and E-cadherin, respectively. Nuclei were stained with 10 µg/mL DAPI (Sigma-Aldrich). Slides were mounted with Prolong Gold (Invitrogen). Images were taken at ×63/1.4 A magnification using a ZEISS LSM 880 Confocal Microscope maintained at the CCAM Microscopy Facility at the University of Connecticut Health Center. Images were captured with *z*-stack and 10% overlap tile scanning to obtain in-depth bacterial localization on each mucosal sample. Finally, images were processed with ImageJ to generate a final image.

### DNA Extraction, 16S rRNA Gene Amplicon Library Construction, and Sequencing

DNA was extracted from whole tissue biopsies (proximal ACF and adjacent normal mucosa) using the MoBio PowerMag Soil 96 well kit (MoBio Laboratories) according to the manufacturer’s protocol for the Eppendorf epMotion liquid-handling robot. DNA extracts were quantified using the Quant-iT PicoGreen kit (Thermo Fisher Scientific). The V4 hypervariable region of the bacterial 16S rRNA gene was amplified using 30 ng of extracted DNA as a template and the primer set of 515 F and 806 R with Golay code indices.^43^ Samples were amplified in triplicate using GoTaq (Promega) with the addition of 10 µg of BSA (New England BioLabs). The PCR reaction was incubated at 95 °C for 3.5 min with 30 cycles of 30 s at 95.0 °C, 30 s at 50.0 °C, and 90 s at 72.0 °C, followed by a final extension at 72.0 °C for 10 min. PCR products were pooled for quantification and visualization using the QIAxcel DNA Fast Analysis (Qiagen). PCR products were normalized based on the concentration of DNA from 250 to 400 bp, then pooled using the QIAgility liquid-handling robot. The pooled PCR products were cleaned up using the Mag-Bind RxnPure Plus (Omega Bio-tek) according to the manufacturer’s protocol. The cleaned pool was sequenced on the MiSeq (Illumina, Inc.) using the v2 2 × 250 base-pair kit (Illumina, Inc.). Positive/negative controls were included for DNA extraction and amplification.

### Sequence Data Processing

Raw 16S rRNA gene reads were processed in mothur.^44^ Sequencing reads were processed by removing the sequences with low quality (average quality < 25) and ambiguous bases (N’s). Chimeric amplicons were removed using UChime software. For 16S, an OTU-based approach was used by clustering sequences with 3% dissimilarity cutoff in Usearch^45^ and the taxonomic classification via the Ribosomal Database Project Classifier.^46^ Shannon diversity index was calculated for each sample and visualized using GraphPad Prism software. Significant separation of microbiome samples based on pairwise thetaYC distance was calculated, visualized as PCoA plots using R. Hierarchical clustering of ACF and normal mucosal bacterial communities based on OTU relative abundances was performed via Jaccard distances and the average linkage method in Morpheus. RAWGraph was utilized to visualize Microbiome Clusters A and B.

### Predictive Functional Profiling

Predictive functional profiling was performed using a phylogenetic investigation of communities by reconstruction of unobserved states (PICRUSt)^47^ inferred from 16S rRNA gene copies as OTUs. Predicted functional gene contents or KEGG Orthologs of bacterial communities were estimated further to predicted functional pathways using “*predict_metagenomes.py”* command of PICRUSt with default parameters. Differentially featured gene function was selected using LEfSe^48^ with the default settings. Results were filtered to contain a logarithmic LDA score >2.5.

### Mutation Analysis

The customized DNA mass spectrometry assay design included the following CRC hotspot somatic mutations: *APC_R1450X*, *APC_S1465fsX3*, *APC_R876X*, *KRAS_G12DV*, *NRAS_G12D*, *NRAS_G13D*, *BRAF_V600E*, *ERBB2_G776insYVMA-c2325*, and *ERBB2_G776insYVMA-c2324*.^9^ Mutation screening was performed on a fee-for-service basis by the Genomics Shared Resources Core facility at the Roswell Park Comprehensive Cancer Center.^49^ Briefly, the protocol involves PCR amplification of DNA using single-nucleotide polymorphism-specific primers, followed by a base-extension reaction using the iPLEX PRO chemistry (Agena Bioscience). The PCR products were treated with (shrimp alkaline phosphatase, then temperature-ramped to 85 °C for 5 min to remove excess dNTPs. iPLEX PRO extension enzyme (Agena) was used for the base-extension reactions. The final base-extension products were treated with SpectroCLEAN (Agena) resin to remove contaminating salts. The extension product was spotted on a 384-pad SpectroCHIP II (Agena) using a Sequenom MassARRAY Nanodispenser (Agena, San Diego, CA). A MassARRAY Analyzer Compact MALDI-TOF MS (Agena) was used for data acquisition. All resultant genotyping calls were performed by the MassARRAY Typer Analyzer v4.0.26.73 (Agena).

### Statistical Analyses

The significance for PCoA plot clustering was tested *via* analysis of molecular variance.^50^ Differentially abundant taxa between ACF and normal mucosa within subject, across subject, and based upon mutation subtypes were identified via Wilcoxon’s signed-rank test or the Mann–Whitney test, as appropriate. All statistical tests were adjusted for multiple comparisons using the Benjamini–Hochberg false discovery rate method. Correlations between the sum of relative abundances of Microbiome Clusters A or B signature OTUs and clinical parameters were performed via Spearman’s rank-order tests.

### Reporting summary

Further information on research design is available in the Nature Research Reporting Summary linked to this article.

## Supplementary information

Supp Info

Reporting summary

## Data Availability

Sequence data that support the findings of this study have been deposited in SRA (PRJNA511474).
