# Supplementary material for: Characterization of Mucosal Dysbiosis of Early Colonic Neoplasia
Source: NPJ Precis Oncol. 2019 Nov 14;3:29. doi: 10.1038/s41698-019-0101-6 (PMC6856115; doi:10.1038/s41698-019-0101-6)
Supplement: Supplementary file 1 — Supp Info [file 41698_2019_101_MOESM1_ESM.docx]

**SUPPLEMENTARY TABLES AND FIGURES**

**Table S1**. Aberrant Crypt Foci (ACF) Distribution by Groups in the Study Population

|  | | Normal | | ACF only | ACF + Polyp |  |
| --- | --- | --- | --- | --- | --- | --- |
| Total ACF | 17.4 ± 16.8 | | 21.1 ± 15.1 | | 21.5 ± 13.7 | |
| Right Side ACF | 0 | | 1.6 ± 1.2 | | 1.4 ± 0.8 | |
| Left Side ACF | 17.4 ± 16.8 | | 19.5 ± 15.3 | | 20.1 ± 13.1 | |
| Number of Polyps | 0 | | 0 | | 2.2 ± 1.0 | |

NOTE: Values are means ± SD

**Table S2**. Location and Pathology Reports of Polyps in Patients on Group 3

| Polyp Location and Pathology | Number of Polyps |  |
| --- | --- | --- |
| Ascending Colon Tubular Adenoma | 13 | |
| Cecum Hyperplastic | 2 | |
| Cecum Sessile Serrated Adenoma | 1 | |
| Cecum Tubular Adenoma | 5 | |
| Descending Colon Tubular Adenoma | 1 | |
| Sigmoid Colon Tubular Adenoma | 6 | |
| Sigmoid Colon Tubulovillous Adenoma | 1 | |
| Sigmoid Hyperplastic | 2 | |
| Transverse Colon Hyperplastic | 1 | |
| Transverse Colon Tubular Adenoma | 1 | |


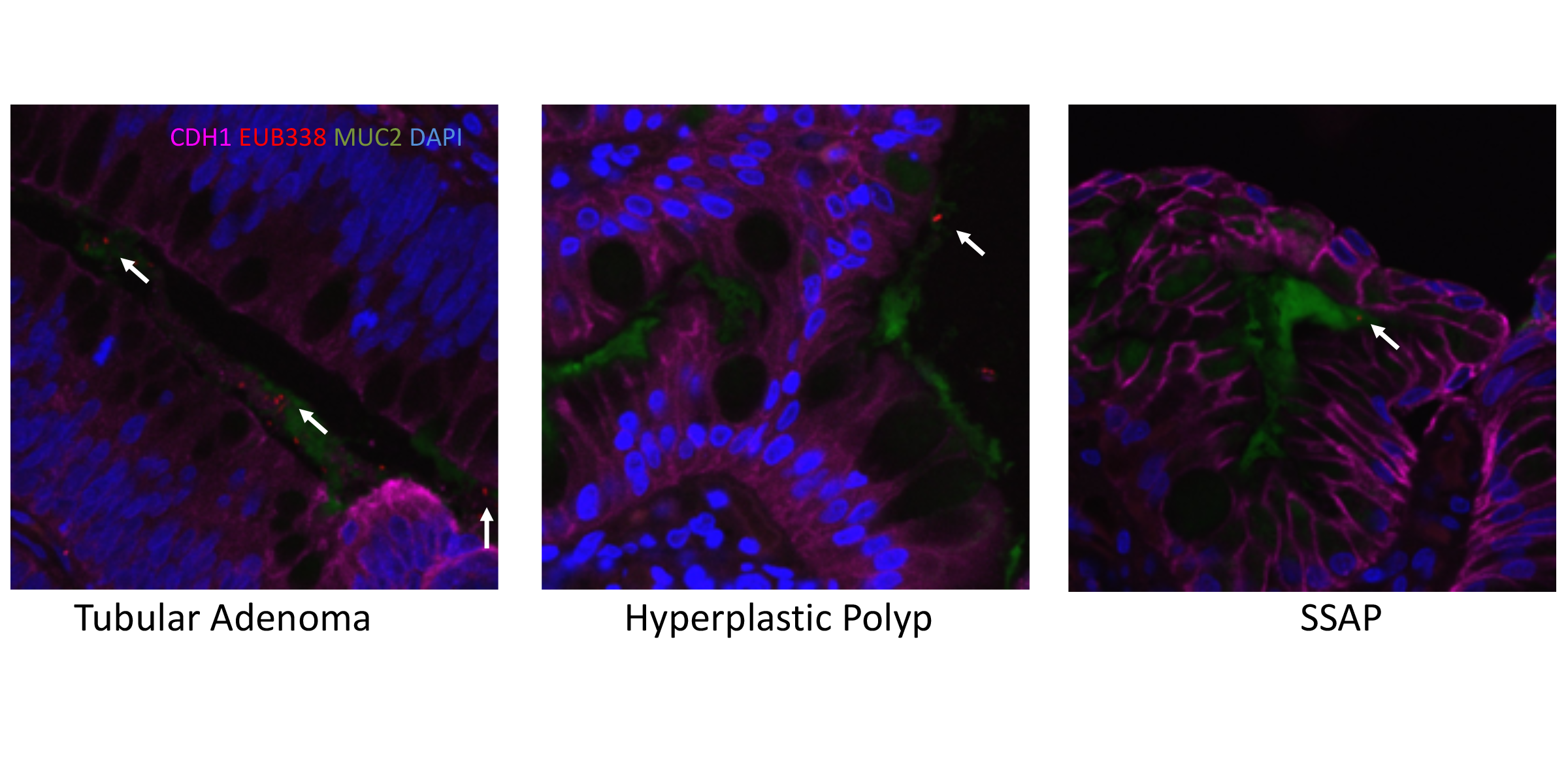


**Figure S1. Direct visualization of colon-associated bacteria present within the colonic mucosa of representative polyp tissues**. Synchronous polyps obtained from patients in Group III were directly examined for the presence of adherent bacteria using 16S universal FISH probes. Red: bacteria (EUB338-cy3 probe), Magenta: E-cadherin, Green; Mucin-2, Blue: DAPI. Bacteria are depicted by white arrows. Bacteria were observed within the mucous layer associated with the epithelium.

**
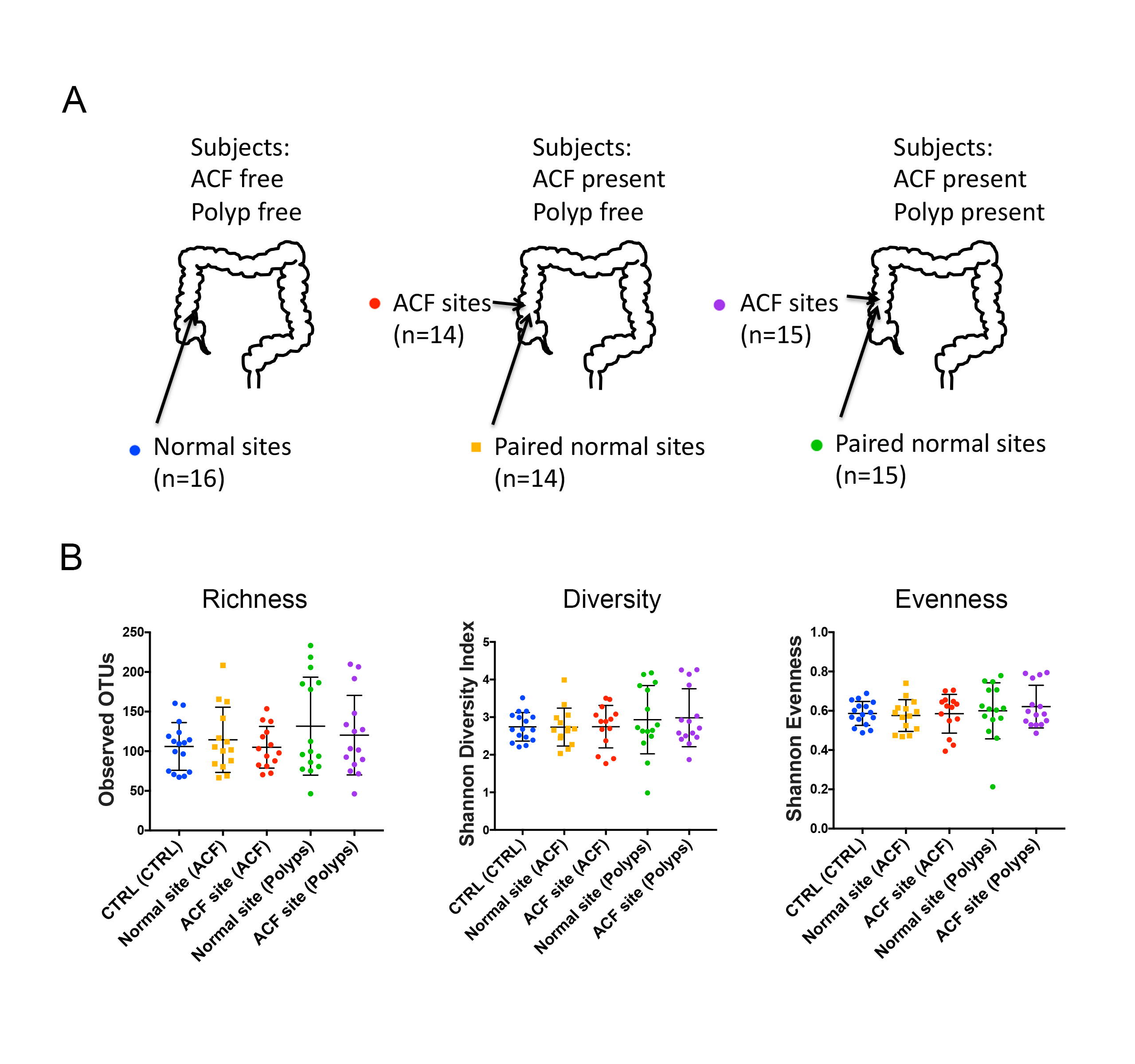
**

**Figure S2. Study design and bacterial diversity comparison by biopsy location**. (A) Normal mucosal samples were taken from ACF-free and polyp-free subjects (blue). ACF lesions were biopsied from polyp-free subjects (red), as well as subjects with proximal polyp(s) present (purple). (B) Paired normal mucosa was biopsied from polyp-free subjects (yellow) and subjects with polyp(s) present in the proximal colon (green) in order to compare the microbiome of different sites within the same subject. No statistical differences were found across all sample sites using measurements of bacterial richness (number of taxa found), evenness and diversity based on Shannon diversity and evenness indices in any pair of comparison groups.

**
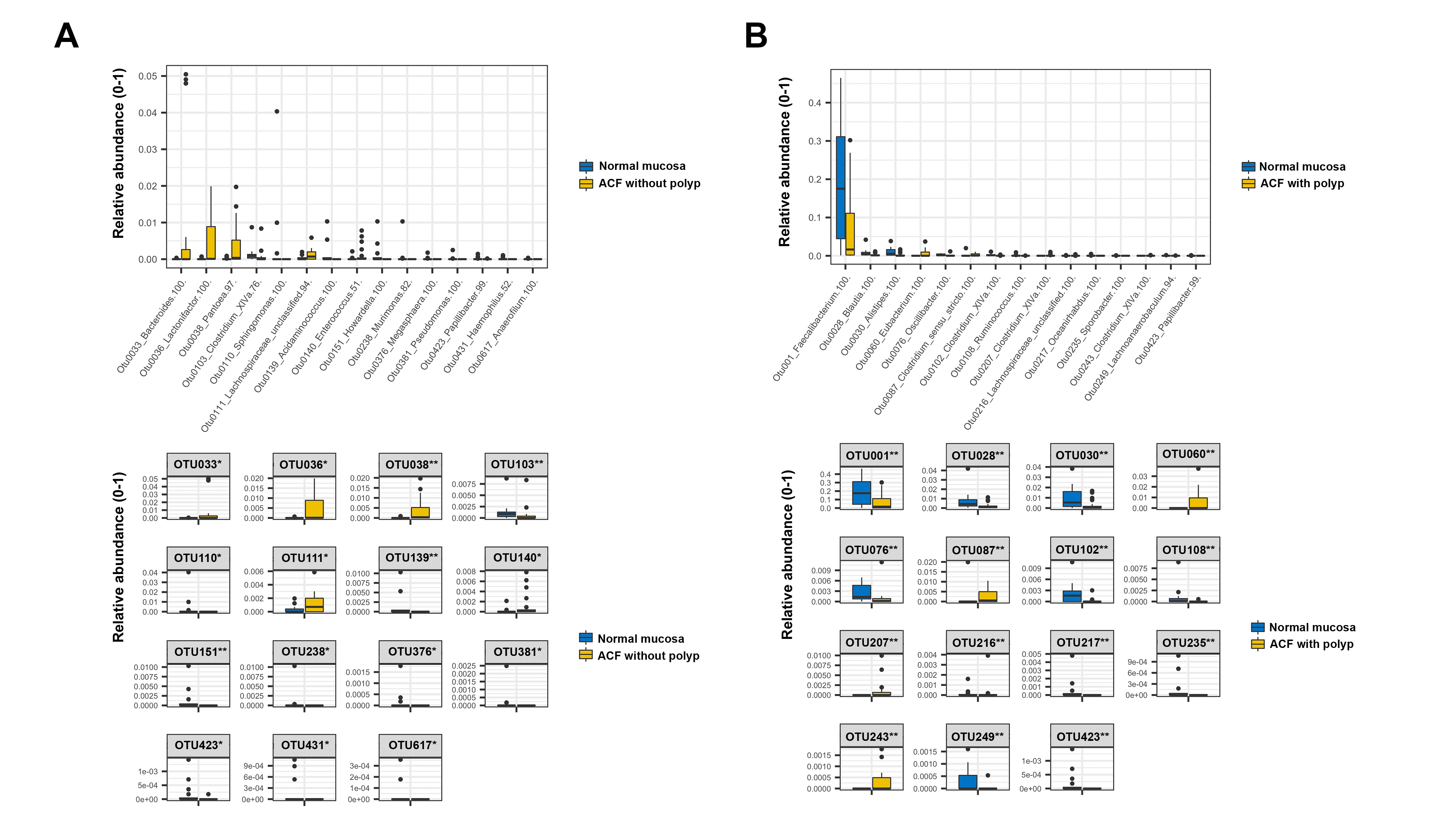
**

**Figure S3.** **Differentially abundant OTUs between ACF, with and without synchronous polyps.** The top 15 differentially abundant OTUs occurring between ACF biopsies without a polyp (left panel) and with a synchronous polyp (right panel) compared to normal mucosa taken from the control subjects. Individual taxa were plotted separately (bottom) for taxa showing significance between ACF and normal mucosa from control subjects using Wilcoxon-signed rank test. *Indicates a p-value less than 0.05, **indicates a p-value less than 0.005

**
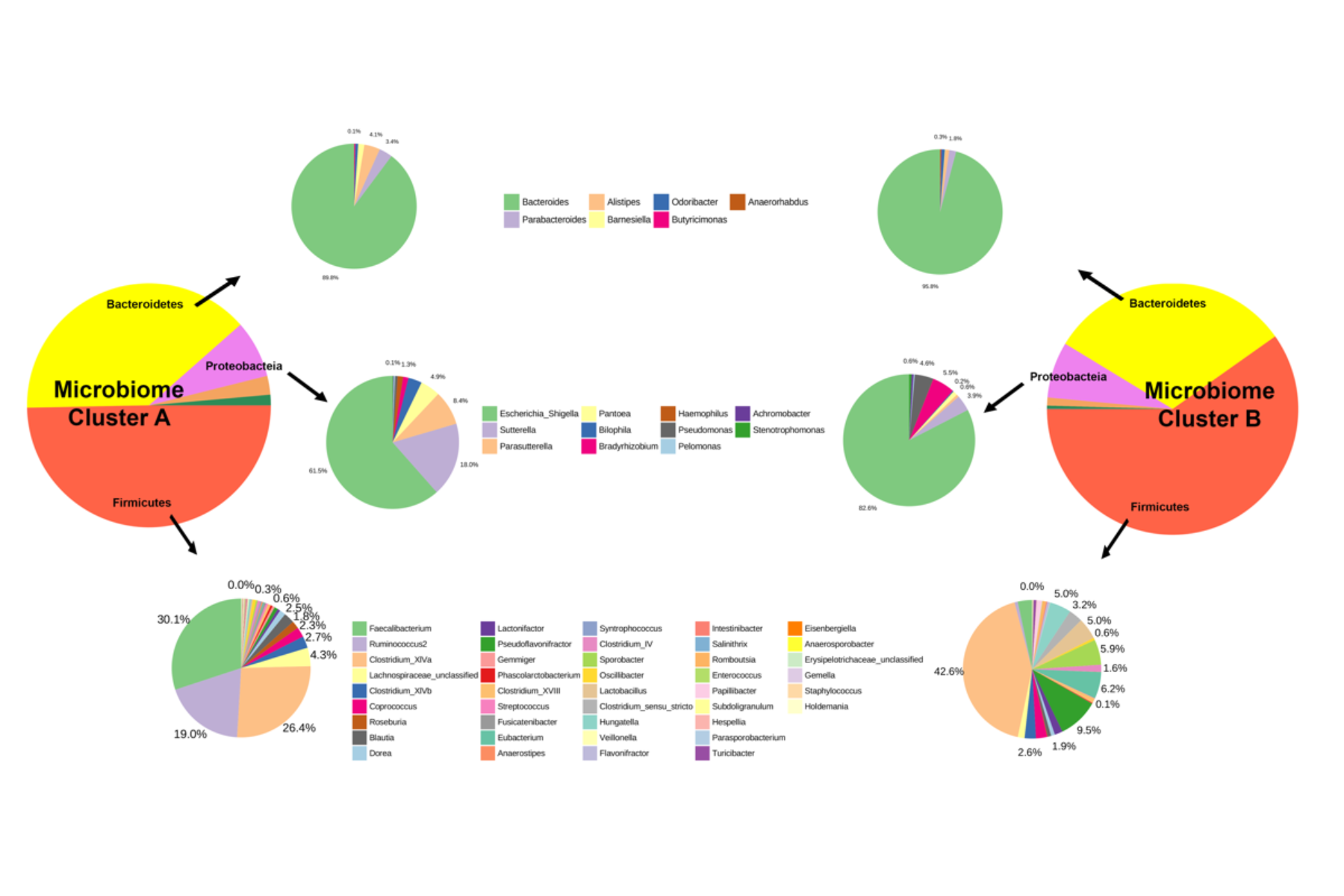
**

**Figure S4.** Top 3 most abundant phyla between Microbiome Clusters A and B were characterized at the genus taxonomic level. Firmicutes, Bacteroidetes and Proteobacteria were the most abundant phyla in both cluster types. The most distinct bacterial composition observed between two clusters was within the Firmicutes phylum. Microbiome Cluster A showed *Faecalibacterium* as a dominant genus, while Microbiome Cluster B showed *Clostridium XIVa* as a dominant genus.
